# New Method for Estimation of Aeolian Sand Transport Rate Using Ceramic Sand Flux Sensor (UD-101)

**DOI:** 10.3390/s91109058

**Published:** 2009-11-13

**Authors:** Keiko Udo

**Affiliations:** Disaster Control Research Center, Tohoku University, 6-6 Aoba, Aoba-ku, Sendai 980-8579, Japan; E-Mail: udo@potential1.civil.tohoku.ac.jp; Tel.: +81-22-795-4843; Fax: +81-22-795-4843

**Keywords:** piezoelectric sensor, impact counts, wind-blown sand transport, instantaneous sand transport, moisture content

## Abstract

In this study, a new method for the estimation of aeolian sand transport rate was developed; the method employs a ceramic sand flux sensor (UD-101). UD-101 detects wind-blown sand impacting on its surface. The method was devised by considering the results of wind tunnel experiments that were performed using a vertical sediment trap and the UD-101. Field measurements to evaluate the estimation accuracy during the prevalence of unsteady winds were performed on a flat backshore. The results showed that aeolian sand transport rates estimated using the developed method were of the same order as those estimated using the existing method for high transport rates, *i.e.*, for transport rates greater than 0.01 kg m^−1^ s^−1^.

## 1. Introduction

Aeolian sand transport is an important process that is studied in many fields such as physics, geosciences, agriculture, and coastal engineering. Considerable effort has been devoted to understanding sand transport mechanisms, including the aerodynamic entrainment of sand grains, acceleration of sand grains in air streams, and transport of sand by steady winds [1–16].

Aeolian sand transport mechanisms observed in the field under unsteady wind conditions differ significantly from those observed under steady wind conditions. Since the 1990s, high-frequency sampling systems such as piezoelectric mass flux sensors [17–22], high-temporal-resolution sediment traps [23–27], and microphonic flux sensors [28] have been employed for the detection of aeolian sand flux at frequencies of several hundreds of hertz and higher. Wind tunnel and field experiments performed using these systems have improved our understanding of sand transport mechanisms under unsteady wind conditions. However, further investigations are required, especially under complex field conditions.

The ceramic sand flux sensor UD-101 (Chuo Kosoku), which is a piezoelectric sand flux sensor that can measure the flux of aeolian sand impacting on its surface, was developed by Kubota *et al.* [29,30] (see Figure 5c). The sensor detects piezoelectric signals generated by collisions between aeolian sand grains and the sensor surface using the same principle as that employed in Sensit [17] and Safires [19]. A significant difference between UD-101 and the other piezoelectric sensors is that UD-101 is unidirectional and does not self-orient toward the wind direction, while both Sensit and Safires are omnidirectional. However, UD-101 has the following advantages over the other piezoelectric sensors:

- The sensor is calibrated by performing both wind tunnel and field experiments.
- The sensor records an impact value of 0 or 1 at 10 kHz; it samples once per second, and theoretically, it can detect a maximum of 10,000 impact counts in the sampling interval, i.e., the sensor has high temporal resolution.
- The sensor is relatively inexpensive.

Udo *et al.* [22] carried out short-term field observations at the Hasaki Beach in Japan using UD-101 during the period 12–16 January 2005. The following features were inferred from the UD-101 data:

- During periods without rainfall and in the presence of longshore winds (conditions similar to those employed in wind tunnel experiments), the aeolian sand flux at a certain height above a flat ground surface increased significantly with the wind velocity and approximately equaled the flux estimated in wind tunnel experiments using Kawamura's [3] equation.
- The flux decreased significantly with an increase in precipitation, i.e., with an increase in the moisture content of the sand surface; however, even during periods with rainfall, flux was detected during strong wind conditions.
- The flux increased with a decrease in the angle between the sensor direction and wind direction.

Further, Udo [31] carried out relatively long-term observations during the period from April 2005 to January 2006 at the Hasaki Beach and showed that a large amount of sand was transported during typhoons. This result implies that UD-101 can be employed for performing long-term flux measurements even during storm events. As the next step toward making long-term flux measurements in fields possible, it is necessary to develop a measurement method for not only the aeolian sand flux at a certain height above the ground surface but also the aeolian sand transport rate (e.g., the total aeolian sand flux between the ground surface and the maximum height of the aeolian sand layer). In particular, the accurate estimation of the rate of seasonal, yearly, or decadal sediment transport due to both littoral drift and aeolian transport at beaches would be invaluable for coastal management. However, the aeolian transport rates estimated by most researchers have not been validated because of the lack of practical estimation/measurement methods; examples of such estimations are estimations made by using conventional equations along with the mean wind data [32] or by integrating areas of beach morphological changes from a boundary under the assumption that the sand transport rate at the boundary is zero [33].

The objective of this study was to develop a new method employing UD-101 for the estimation of the aeolian sand transport rate [29,30]. We applied the method to field data obtained during the period from 22 September to 6 December 2005 [31] and assessed the estimation accuracy to examine the suitability of the method for the direct estimation of long-term transport rates in the field.

## 2. New Estimation Method for Determining Total Aeolian Sand Flux

### 2.1. Characteristics of UD-101 Data

**Figure 1.**
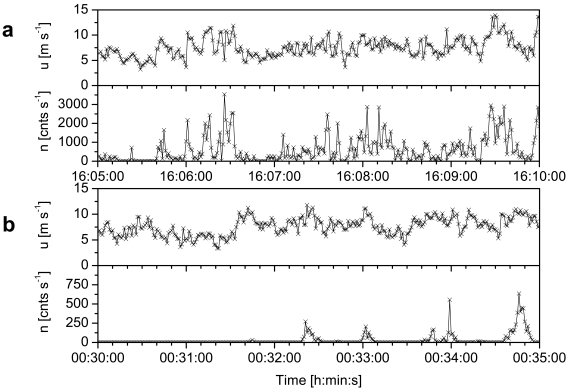
Time series of instantaneous horizontal wind velocity *u* and blown-sand impact count *n* at the Hasaki Beach [22,31]; the data were recorded for 5 min at a frequency of 1 Hz (a) during intervals without rainfall on 12 January 2005 and (b) after a rainfall event on 25 September 2005. The wind velocity was measured using an ultrasonic anemometer at a height of 0.9 m above the flat ground surface (*z_u_* = 0.9 m). The impact count was measured using UD-101 at *z_s_* = 0.04 m. The horizontal distance between the wind and saltation sensors was 1 m. The median sand grain size *d*_50_ of the ground surface at the beach was 0.2 × 10^−3^ m.

Figure 1 shows a time series of the instantaneous horizontal wind velocity *u* and blown-sand impact count *n* measured at the Hasaki Beach; the median sand grain size *d*_50_ at the beach was 0.2 × 10^−3^ m [22,31]. *n* shows a positive relationship with *u* during periods without any rainfall, but decreases significantly after a rainfall event. The sensor cannot detect the impact of blown sand if wet sand adheres to its surface after a rainfall event; however, it can detect the impact if this sand is subsequently removed by heavy rainfall. The sensor does not detect the impact of raindrops.

Many studies have shown that the vertical wind velocity profile in a wind tunnel is logarithmic [34,35]. In previous studies, the threshold wind velocity *u_t_* has been estimated from both a wind profile law and Bagnold's [1] equation. The logarithmic wind profile law is expressed as:

(1) $$u=\frac{u_{*}}{\kappa }\ln\left(z_{u}/z_{0s}\right)$$

Here, *u*∗ is the wind shear velocity, *κ* is the von Kármán constant (= 0.4), *z_u_* is the height at which *u* is measured, and *z*_0_*_s_* is the saltation roughness length. Saltation is the hopping motion of sand grains and the primary mode of aeolian sand transport in beach intertidal and supratidal zones. Aeolian sand transport leads to an increase in the aerodynamic roughness length. *u_t_* is obtained by substituting *u*∗ = *u*∗*_t_* in Equation (1). The threshold wind shear velocity *u*∗*_t_* is estimated from Bagnold's [1] equation:

(2) $$u_{*t}=a\sqrt{\frac{\sigma -\rho }{\rho }gd}$$

where *a* is the numerical transport rate coefficient (= 0.1), *ρ* is the air density, *σ* is the sand density (= 2,650 kg m^−3^), *g* is the gravitational acceleration, and *d* is the sand grain size. *z*_0_*_s_* is estimated from the relation proposed by Charnock [36] and Chamberlain [37]:

(3) $$z_{0s}=c_{0}\frac{{u_{*}}^{2}}{2g}$$

where *c*_0_ is a constant (= 0.16 for saltation on beaches [38]).

### 2.2. Relationship between Impact Counts and Aeolian Sand Flux at a Measurement Height

Kubota *et al.* [30] performed experiments in a 20-m-long wind tunnel using the experimental setup that was employed by Hotta *et al.* [39]; the sand used in the experiments had a *d*_50_ value of 0.25 × 10^−3^ m. The authors showed that the impact counts approached a constant value asymptotically at frequencies greater than 8 kHz. They also compared the sand mass flux *q_s_* measured in the wind tunnel using an 8-kHz sensor [30] with *q* measured using a vertical-distribution-type sand trap [39]. *q_s_* at the measurement height *z_s_* was estimated from *n* using the following equation [22]:

(4) $$q_{s}\left(z_{s}\right)=\frac{2\sigma {d_{50}}^{3}n}{3{d_{s}}^{2}t_{0}}$$

where *t*_0_ is the sampling period of *n* (= 1 s) and *d_s_* is the diameter of the sensor (= 0.012 m). The vertical-distribution-type sand trap was developed by Hotta and Horikawa [40], and it can be used to measure the flux at 37 different heights in the range 0–0.505 m. *q* was corrected for trap efficiency. The wind tunnel experiments performed by Kubota *et al.* [30] and Hotta *et al.* [39] showed that *q_s_*(*z_s_*) for *d*_50_ = 0.25 × 10^−3^ m was related to *q*(*z_s_*) measured using the vertical-distribution-type sand trap [22], which was closely approximated as:

(5) $$q\left(z_{s}\right)=10.57q_{s}\left(z_{s}\right)$$

### 2.3. Relationship between Aeolian Sand Flux at a Measurement Height and Total Sand Flux

**Figure 2.**
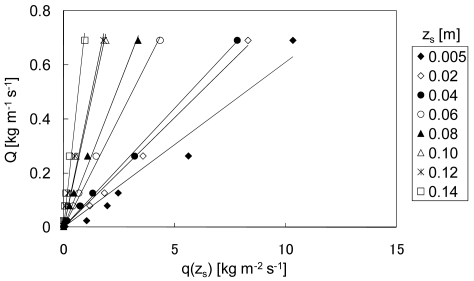
Relationship between the sand flux at *z_s_, q*(*z_s_*), and the total sand flux *Q* for *d*_50_ = 0.25 × 10^−3^ m.

**Figure 3.**
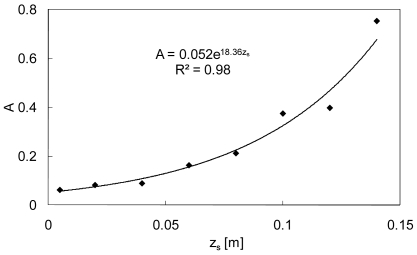
Relationship between *z_s_* and *A* for *d*_50_ = 0.25 × 10^−3^ m.

The relationship between the sand flux at *z_s_, q*(*z_s_*) [kg m^−2^ s^−1^], and the total sand flux *Q* [kg m^−1^ s^−1^] is shown in Figure 2. Hotta *et al.* [39] employed the same wind tunnel as that used by Kubota *et al.* [30], with 0.17 m s^−1^ < *u*∗ < 1.77 m s^−1^ and 0.14 × 10^−3^ m < *d*_50_ < 0.68 × 10^−3^ m. Here, only the data for *d*_50_ = 0.25 × 10^−3^ m are shown. *Q* was found to be approximately proportional to *q*(*z_s_*), as expressed by:

(6) $$Q=Aq\left(z_{s}\right)$$

where the regression coefficient *R*^2^ of the approximation was 0.96, 0.99, 1.00, 1.00, 0.99, 0.98, 0.97, and 0.97 for *z_s_* = 0.005, 0.02, 0.04, 0.06, 0.08, 0.10, 0.12, and 0.14 m, respectively. As shown in Figure 3, the constant *A* shows a positive relationship with *z_s_*, and it can be expressed as:

(7) $$A=B\exp\left(Cz_{s}\right)$$

The relationships between *z_s_* and *R*^2^ and between *z_s_* and *A* (shown in Figures 2 and 3, respectively) indicate that *z_s_* should be set in the range 0.02–0.08.

**Figure 4.**
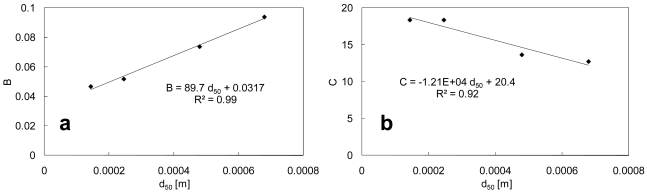
Relationship between **(a)** *d*_50_ and *B* and **(b)** *d*_50_ and *C*.

The constants *B* and *C* show a positive relationship with *d*_50_ (Figure 4):

(8) $$B=89.7d_{50}+0.0317$$

and:

(9) $$C=-1.21\times 10^{4}d_{50}+20.4$$

Finally, the following equation is derived from Equations (4)–(9):

(10) $$Q=\frac{7.05\sigma {d_{50}}^{3}n}{{d_{s}}^{2}t_{0}}\left(89.7d_{50}+0.0317\right)\exp\left[\left(-1.21\times 10^{4}d_{50}+20.4\right)z_{s}\right]$$

This equation suggests that *Q* can be estimated only if *n, d*_50_, and *z_s_* are measured by performing field experiments, although the equation was derived from observations made during 20-m-long wind tunnel experiments performed by Hotta *et al.* [39] and Kubota *et al.* [30] on a flat ground surface under steady wind conditions with *u*∗ < 1.8 m s^−1^ and at a sensor height of 0.005 < *z_s_* < 0.14.

## 3. UD-101 Data Obtained at Hasaki Beach

### 3.1. Summary of Field Measurements

**Figure 5.**
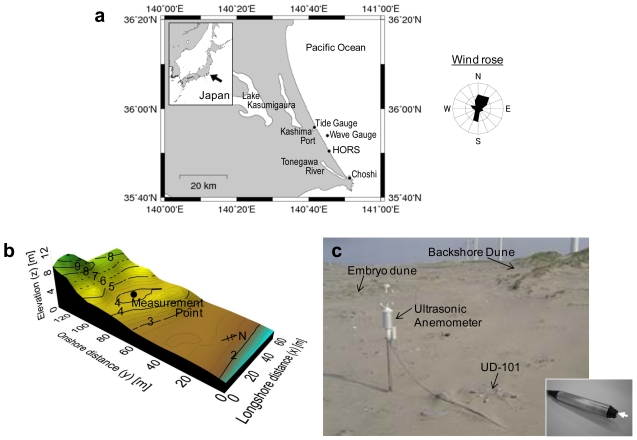
(a) Location of the study site (Hasaki Oceanographical Research Station, HORS) and the wind rose for 2004. (b) Topography of the Hasaki Beach and the location of the measurement point. The cross-shore distance to the average annual shoreline is 0 m; the offshore direction was considered positive. (c) Location of the measurement point on 17 January 2005. The ultrasonic anemometer and ceramic sand flux sensors were installed as shown. The four UD-101 sensors were oriented toward the north, east, south, and west directions.

Field measurements were performed at the Hasaki Beach using UD-101. The beach faces the Pacific Ocean and is located in Ibaraki Prefecture [35°50′25″N, 140°45′42″E; Figure 5(a)] [22,31]. The beach is of the dissipative type and has a foreshore slope ranging from 0.02 to 0.05 and a width of 100 m [Figure 5(b)]. *d*_50_ is approximately 0.2 × 10^−3^ m along the backshore. Beach grasses grow around a small embryo dune at *y* = 50 m and around a longshore dune at *y* = 70 to 90 m just in front of the coastal forest area, where *y* is the onshore distance from the average shoreline position in 2004. The measurement point was located in a flat area between the embryo dune and backshore dune.

The annual wind direction is predominantly in the north-northeast direction [Figure 5(a)]. Winds are relatively weak in summer, except during typhoons, but strong in winter due to the presence of low-pressure systems. *u*, the wind direction *θ*, and *n* [the same data obtained by Udo [31], Figure 5(c)] were measured from 21 September to 3 December 2005. *u* and *θ* were measured at a height of 0.9 m above the measurement point (*z* = 0.9 m) using a three-axis ultrasonic anemometer (Delta Ohm; model: HD2003), while *n* was measured at *z* = 0.04 m using UD-101. Four UD-101 sensors were deployed, and they were directed toward the north, east, south, and west. The wind direction in the horizontal plane is expressed clockwise from north, from 0° to 360°. The data of *u, θ*, and *n* were recorded on a data logger system (FieldPoint cFP-2000, National Instruments) at a frequency of 1 Hz and were automatically downloaded on a weekly basis from the logger. The precipitation *Pr* was measured at 10-min intervals at the Choshi Meteorological Observatory (35°44′18″N, 140°51′24″E) by the Japan Meteorological Agency.

In addition, in this study, *z_s_* was measured manually almost every day in order to calculate *Q* using Equation (10). The instantaneous *z_s_* was calculated by linear interpolation. The *z_s_* value of the sensor oriented toward the wind direction tended to be large and that of the sensor directed in the opposite direction tended to be small; for example, for north winds, the *z_s_* value of the sensor directed northward tended to be large and that of the sensor directed southward tended to be small.

**Figure 6.**
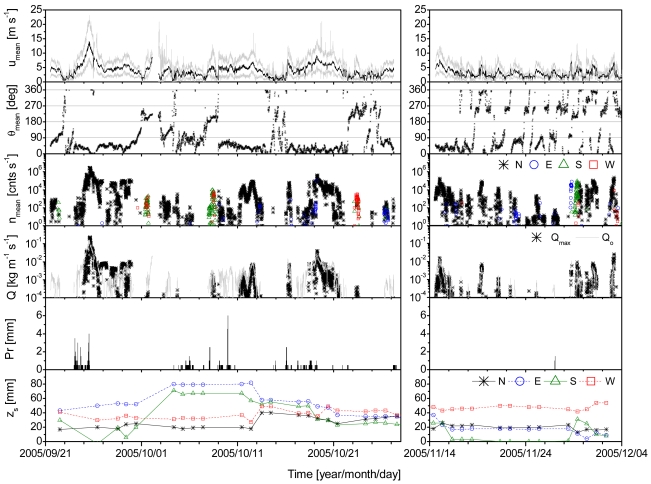
Time series of 10-min averages of the horizontal wind velocity *u_mean_*, horizontal wind direction *θ_mean_*, blown-sand impact count *n_mean_*, estimated total aeolian sand flux *Q_mean_*, and precipitation *Pr*; the time series of *Q_O_* estimated from Equation (13) and the daily sensor height from the ground surface *z_s_* are also shown. The solid gray lines in the top figure show the maximum and minimum instantaneous wind velocities. The wind direction is expressed clockwise from north, from 0° to 360°. Only the maximum *n_mean_* for the four directions in each of the 10-min intervals is plotted.

The measurement results are shown in Figure 6. Data could not be obtained from 28 October to 13 November 2005 because of network problems. Large amounts of sand were transported during the transport events on 24–26 September due to the impact of typhoon 0517, which resulted in a total precipitation of 39.5 mm; similar events involving the transport of a large amount of sand occurred on 11–12 October (period without rainfall); on 18–20 October (due to the impact of typhoon 0520), when the total precipitation was 6 mm; and on 14–15 November (period without rainfall) [31].

### 3.2. Evaluation of the Accuracy of the New Method by Comparing Q with Q_B_, Q_K_, Q_O_, and Q_L_

The *Q* value estimated from *n* was compared with that obtained using the conventional equations for a constant wind velocity in order to examine the accuracy of *Q* estimated from the UD-101 data. Here, the commonly employed equations of Bagnold [1], Kawamura [3], Owen [5], and Lettau and Lettau [6] (for *Q_B_, Q_K_, Q_O_*, and *Q_L_*, respectively) were used [13,16,41–43]:

(11) $$Q_{B}=c_{B}\sqrt{\frac{d}{D}}\frac{\rho }{g}u_{*}^{3}$$

(12) $$Q_{K}=c_{K}\frac{\rho }{g}\left(u_{*}-u_{*t}\right){\left(u_{*}+u_{*t}\right)}^{2}$$

(13) $$Q_{O}=c_{O}\frac{\rho }{g}u_{*}\left(u_{*}-u_{*t}\right)\left(u_{*}+u_{*t}\right)$$

(14) $$Q_{L}=c_{L}\sqrt{\frac{d}{D}}\frac{\rho }{g}u_{*}^{2}\left(u_{*}-u_{*t}\right)$$

Here, *D* is the reference sand grain diameter (= 0.25 × 10^−3^ m) and *c_B_, c_K_, c_O_*, and *c_L_* are constants with values of 1.8, 2.78, 0.25 + *w*_0_/3*u*∗ (where *w*_0_ is the fall velocity), and 4.2, respectively. The sand transport rate was estimated to be zero when *u*∗ < *u*∗*_t_*. *u*∗ was estimated from Equation (1) using the measured value of *u; u*∗*_t_* was obtained using Equation (2) and *w*_0_ was determined from Rubey's [44] equation:

(15) $$w_{0}=\sqrt{\left(s-1\right)gd}\left(\sqrt{\frac{2}{3}+\frac{36\nu^{2}}{\left(s-1\right)gd^{3}}}-\sqrt{\frac{36\nu^{2}}{\left(s-1\right)gd^{3}}}\right)$$

where *s* is the relative density of the sand (= *σ*/*ρ*) and *ν* is the kinematic viscosity of air. These equations were derived for a constant wind velocity, and hence, *Q* estimated using the UD-101 data was averaged over 10 min. The 10-min mean sand flux *Q_mean_* during periods without rainfall was compared to *Q_B_, Q_K_, Q_O_*, and *Q_L_*.

**Figure 7.**
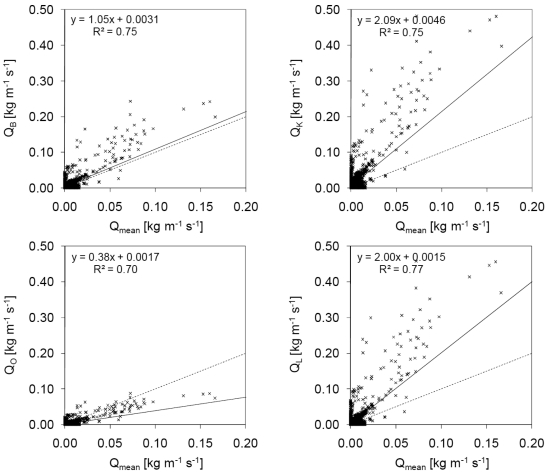
Comparison of *Q_mean_* estimated from *n* with *Q_B_, Q_K_, Q_O_*, and *Q_L_* estimated using the equations given in the text, when *n* ≠ 0 or *u*∗ > *u*∗*_t_* (*i.e., u* > 4.05 m s^−1^) during periods without rainfall. The solid line is the linear regression line. The dashed line is the line that would be obtained in case of perfect agreement.

Figure 7 shows a comparison of *Q_mean_* values obtained from *n* with *Q_B_, Q_K_, Q_O_*, and *Q_L_* values derived from the equations given above, for periods without rainfall. Data for *n* = 0 and *u*∗ < *u*∗*_t_* (i.e., *u* < 4.05 m s^−1^) were excluded. *Q_mean_* showed a clear positive relationship with *Q_B_, Q_K_, Q_O_*, and *Q_L_*, and the estimated values followed the order *Q_O_* < *Q_B_* < *Q_L_* < *Q_K_*. The best agreement was observed between *Q_mean_* and *Q_O_* when 0.02 < *Q_mean_* < 0.10 and between *Q_mean_* and *Q_B_* when *Q_mean_* > 0.10.

**Figure 8.**
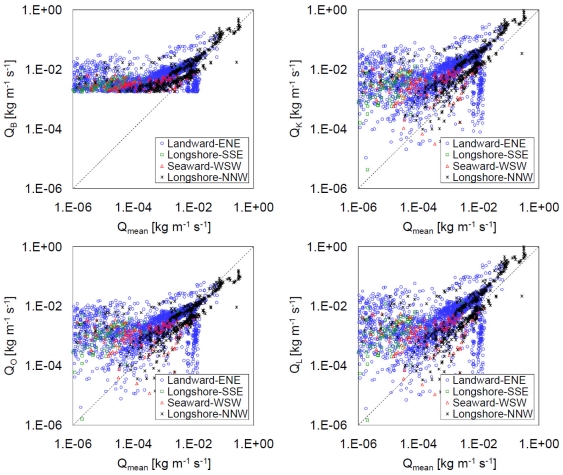
Comparison of *Q_mean_* estimated from *n* with *Q_B_, Q_K_, Q_O_*, and *Q_L_* for east-northeast (15° < *θ_mean_* < 105°), south-southeast (105° < *θ_mean_* < 195°), west-southwest (195° < *θ_mean_* < 285°), and north-northwest (285° < *θ_mean_* < 375°) winds. *Q_B_* is either zero or greater than 1.76 × 10^-3^ kg m^−1^ s^−1^ because Equation (11) does not include the term (*u*∗ – *u*∗*_t_*). The dashed line denotes perfect agreement.

Figure 8 shows double logarithmic plots of *Q_mean_* versus *Q_B_, Q_K_, Q_O_*, and *Q_L_* for east-northeast (15° < *θ_mean_* < 105°), south-southeast (105° < *θ_mean_* < 195°), west-southwest (195° < *θ_mean_* < 285°), and north-northwest (285° < *θ_mean_* < 375°) winds. For small *Q_mean_* values, i.e., less than 0.01 kg m^−1^ s^−1^, the *Q_mean_* value was overestimated by *Q_B_, Q_K_, Q_O_*, and *Q_L_*. The probable causes for the overestimation are the low measurement accuracy of *n* for small *Q_mean_*, the low estimation accuracy of *z*_0s_ for small *u*∗, variations in the wind velocity, and the high moisture content at the beach surface. Raupach [38] showed that *c*_0_ in Equation (3) depended on *u*∗, especially for small *u*∗. A decrease in the instantaneous wind velocity below the threshold velocity, even when the mean wind velocity is greater than the threshold velocity, can lead to an overestimation. The moisture content of the backshore is nonzero even during periods without rainfall because of the proximity of the backshore to the sea. Udo *et al.* [22] demonstrated that the sand transport flux at a height, *q_mean_*, decreased by more than one order of magnitude when the moisture content increased during rainfall periods.

Table 1 lists the linear regression relationships between *Q_mean_* estimated from *n* and *Q_B_, Q_K_, Q_O_*, and *Q_L_* for the east-northeast, south-southeast, west-southwest, and north-northwest winds (see Figure 8). While a strong correlation was observed for the north-northwest wind (in the longshore direction), a clear correlation was not observed in the case of the other winds because only a small number of *Q_mean_* values were greater than 0.01 kg m^−1^ s^−1^. Udo [31] showed the dependency of *q_mean_* on *θ_mean_* and that *q_mean_* was high when *θ_mean_* was in the longshore direction (especially in winter); however, *q_mean_* was low when *θ_mean_* was seaward (in late summer and winter) and was even lower when *θ_mean_* was landward (in spring and autumn). The differences between *Q_mean_* and *Q_B_, Q_K_, Q_O_*, and *Q_L_* were found to be independent of *θ_mean_*.

Hotta and Horikawa [40] performed field experiments at beaches by using a trench-type trap and a vertical-distribution-type trap to estimate the aeolian sand transport rate and showed that the possible ranges of *c_B_* and *c_K_* are 0.5−2.0 and 1.0–3.0, respectively. This result suggests that the actual values of *Q_B_* and *Q_K_* are possibly one-third of those shown in Figures 7 and 8. All the field results showed that the sand transport rate estimated using the new method together with the UD-101 data was of the same order as that estimated using the conventional equations, although several differences were observed between the aeolian sand transport rate in the wind tunnel and that in field experiments [38,40,45,46]. The new method is useful for the direct estimation of the sand transport rate in a field analysis. Further analysis using the UD-101 data can provide additional insights into aeolian sand transport mechanisms in fields.

## 4. Conclusions

In this study, we developed a new method for the estimation of the aeolian sand transport rate and demonstrated the usefulness of UD-101 for field measurements of the aeolian sand transport rate. The transport rates estimated from the UD-101 data were reasonable and showed a clear positive relationship with the rates estimated using the conventional equations. Further, for *Q_mean_* > 0.01 kg m^−1^ s^−1^, the transport rates estimated using the UD-101 data were of the same order as those estimated using the conventional equations. The best agreement was observed between *Q_mean_* and *Q_O_* when 0.02 < *Q_mean_* < 0.10 and between *Q_mean_* and *Q_B_* when *Q_mean_* > 0.10. It is necessary to develop a method for accurately determining the constant values in the conventional equations. The cause for the disagreement between *Q_mean_* and *Q_B_, Q_K_, Q_O_*, and *Q_L_* for small *Q_mean_* should be investigated in a future research. In field analyses, the estimation of large transport rates under strong winds is important and the proposed estimation method is expected to be useful in obtaining the transport rate.

## Figures and Tables

**Table 1.** Linear regression expressions for the relationships between *Q_mean_* estimated from *n*, and *Q_B_, Q_K_, Q_O_*, and *Q_L_* for east-northeast, south-southeast, west-southwest, and north-northwest winds. *R*^2^ is the regression coefficient and *N* is the number of data points. See Figure 8 for related plots.

	**ENE (*N* = 2714)**	**SSE (*N* = 198)**	**WSW (*N* = 557)**	**NNW (*N* = 706)**
*Q_B_*	1.231*Q_mean_* + 0.003 (*R*^2^ = 0.33)	−0.242*Q_mean_* + 0.002 (*R*^2^ = 0.08)	−0.180*Q_mean_* + 0.003 (*R*^2^ = 0.01)	1.047*Q_mean_* + 0.004 (*R*^2^ = 0.82)
*Q_K_*	2.548*Q_mean_* + 0.004 (*R*^2^ = 0.34)	−0.225*Q_mean_* + 0.002 (*R*^2^ = 0.03)	−0.304*Q_mean_* + 0.005 (*R*^2^ = 0.01)	2.075*Q_mean_* + 0.008 (*R*^2^ = 0.82)
*Q_O_*	0.545*Q_mean_* + 0.001 (*R*^2^ = 0.32)	−0.076*Q_mean_* + 0.001 (*R*^2^ = 0.03)	−0.079*Q_mean_* + 0.001 (*R*^2^ = 0.01)	0.369*Q_mean_* + 0.002 (*R*^2^ = 0.81)
*Q_L_*	2.091*Q_mean_* + 0.001 (*R*^2^ = 0.34)	−0.094*Q_mean_* + 0.001 (*R*^2^ = 0.02)	−0.200*Q_mean_* + 0.003 (*R*^2^ = 0.01)	1.997*Q_mean_* + 0.003 (*R*^2^ = 0.82)

## References

[1] Bagnold R.A. (1941). The Physics of Blown Sand and Desert Dunes.

[2] Chepil W. (1945). Dynamics of wind erosion. II: initiation of soil movement. Soil Sci..

[3] Kawamura R. (1951). Study of sand movement by wind. Univ. Tokyo, Rep. Inst. Sci. Technol..

[4] Zingg A.W., McNown J.S., Boyer M.C. (1953). Wind tunnel studies of the movement of sedimentary material.

[5] Owen P.R. (1964). Saltation of uniform grains in air. J. Fluid Mech..

[6] Letau K., Lettau H.H., Lettau H.H., Lettau K. (1978). Experimental and micrometeorological field studies of dune migration. Exploring the World's Driest Climates.

[7] White B.R. (1979). Soil transport by winds on Mars. J. Geophys. Res..

[8] Iversen J.D., White B.R. (1982). Saltation threshold on Earth, Mars and Venus. Sedimentology.

[9] Unger J.E., Haff P.K. (1987). Steady state saltation in air. Sedimentology.

[10] Werner B.T. (1990). A steady-state model of wind-blown sand transport. J. Geol..

[11] Anderson R.S., Haff P.K. (1991). Wind modification and bed response during saltation of sand in air. Acta Mech..

[12] Shao Y., Raupach M.R. (1992). The overshoot and equilibrium of saltation. J. Geophys. Res..

[13] Iversen J.D., Rasmussen K.R. (1999). The effect of wind speed and bed slope on sand transport. Sedimentology.

[14] Shao Y., Lu H. (2000). A simple expression for wind erosion threshold friction velocity. J. Geophys. Res..

[15] Roney J.A., White B.R. (2004). Definition and measurement of dust aeolian thresholds. J. Geophys. Res..

[16] Sorensen M. (2004). On the rate of aeolian sand transport. Geomorphology.

[17] Stockton P.H., Gillette D.A. (1990). Field measurement of the sheltering effect of vegetation on erodible land surfaces. Land Degrad. Rehabil..

[18] Stout J.E., Zobeck T.M. (1997). Intermittent saltation. Sedimentology.

[19] Baas A.C.W. (2004). Evaluation of saltation flux impact responders (Safires) for measuring instantaneous aeolian sand transport intensity. Geomorphology.

[20] Gillies J.A., Berkofsky L. (2004). Eolian suspension above the saltation layer, the concentration profile. J. Sediment. Res..

[21] Mikami M., Shi G.Y., Uno I., Yabuki S., Iwasaka Y., Yasui M., Aoki T., Tanaka T.Y., Kurosaki Y., Masuda K., Uchiyama A., Matsuki A., Sakai T., Takemi T., Nakawo M., Seino N., Ishizuka M., Satake S., Fujita K., Hara Y., Kai K., Kanayama S., Hayashi M., Du M., Kanai Y., Yamada Y., Zhang X.Y., Shen Z., Zhou H., Abe O., Nagai T., Tsutsumi Y., Chiba M., Suzuki J. (2006). Aeolian dust experiment on climate impact: an overview of Japan-China joint project ADEC. Global Planet. Change..

[22] Udo K., Kuriyama Y., Jackson D.W.T. (2008). Observations of wind-blown sand under various meteorological conditions. J. Geophys. Res..

[23] Butterfield G.R. (1991). Grain transport rates in steady and unsteady turbulent airflows. Acta Mech..

[24] Butterfield G.R. (1998). Traditional behaviour of saltation: wind tunnel observations of unsteady winds. J. Arid Environ..

[25] Jackson D.W.T. (1996). A new, instantaneous aeolian sand trap design for field use. Sedimentology.

[26] Bauer B.O., Namikas S.L. (1998). Design and field test of a continuously weighing, tipping-bucket assembly for aeolian sand traps. Earth Surf. Process. Landforms.

[27] Davidson-Arnott R.G.D., MacQuarrie K., Aagaard T. (2005). The effect of wind gusts, moisture content and fetch length on sand transport on a beach. Geomorphology.

[28] Ellis J.T., Morrison R.F., Priest B.H. (2009). Detecting impacts of sand grains with a microphone system in field conditions. Geomorphology.

[29] Kubota S., Hosaka K., Oguri Y. (2006). Development of wind blown sand measuring device used a ceramic piezo-electric sensor: Output of a ceramic piezo-electric sensor when sand grains hit. Rep. Res. Inst. Sci. Technol. Nihon Univ..

[30] Kubota S., Hosaka K., Tamura T. (2007). Development of wind blown sand measuring device used a ceramic piezo-electric sensor. (2) Verification by a visual analysis using a high-speed camera. Rep. Res. Inst. Sci. Technol. Nihon Univ..

[31] Udo K. (2009). Field measurement of seasonal wind-blown sand flux using high-frequency sampling instrumentation. J. Coast. Res..

[32] U.S. Army Corps of Engineers (2008). Coastal Engineering Manual 1110-2-1100.

[33] Kuriyama Y., Mochizuki N., Nakashima T. (2005). Influence of vegetation on aeolian sand transport rate from a backshore to a foredune at Hasaki, Japan. Sedimentology.

[34] Ni J.R., Li Z.S., Mendoza C. (2002). Vertical profiles of aeolian sand mass flux. Geomorphology.

[35] Rasmussen K.R., Sørensen M. (2008). Vertical variation of particle speed and flux density in aeolian saltation: measurement and modeling. J. Geophys. Res..

[36] Charnock H. (1955). Wind stress on a water surface. Q. J. R. Meteorol. Soc..

[37] Chamberlain A.C. (1983). Roughness length of sea, sand, and snow. Boundary Layer Meteorol..

[38] Raupach M.R. (1991). Rough-wall turbulent boundary layers. Appl. Mech. Rev..

[39] Hotta S., Kubota S., Nakamura N., Hosaka K., Smith J.M. (2007). Wind tunnel study of vertical distribution of sand transport rate by wind.

[40] Hotta S., Horikawa K. (1991). Vertical distribution of sand transport rate by wind. Coast. Eng. J..

[41] Sherman D.J., Jackson D.W.T., Namikas S.L., Wang J. (1998). Wind-blown sand on beaches: An evaluation of models. Geomorphology.

[42] Shao Y. (2000). Physics and Modelling of Wind Erosion.

[43] Ni J.R., Li Z.S., Mendoza C. (2004). Blown-sand transport rate. Earth Surf. Process. Landf..

[44] Rubey W.W. (1933). Settling velocities of gravel, sand, and silt particles. Am. J. Sci..

[45] Horikawa K., Hotta S., Kubota S., Katori S. (1983). On the sand transport rate by wind on a beach. Coast. Eng. Japan.

[46] Sherman D.J., Farrell E.J. (2008). Aerodynamic roughness lengths over movable beds: Comparison of wind tunnel and field data. J. Geophys. Res..

